# The relationships between exogenous and endogenous antioxidants with the lipid profile and oxidative damage in hemodialysis patients

**DOI:** 10.1186/1471-2369-12-59

**Published:** 2011-10-26

**Authors:** Miguel Roehrs, Juliana Valentini, Clóvis Paniz, Angela Moro, Mariele Charão, Rachel Bulcão, Fernando Freitas, Natália Brucker, Marta Duarte, Mirna Leal, Geni Burg, Tilman Grune, Solange Cristina Garcia

**Affiliations:** 1 Laboratory of Toxicology (LATOX), Department of Clinical and Toxicology, Faculty of Pharmacy, Federal University of Rio Grande do Sul, Porto Alegre, RS, Brazil; 2 Post-graduate Program in Pharmaceutical Sciences, Federal University of Rio Grande do Sul, Porto Alegre, RS, Brazil; 3 Departament of Physiology, Federal University of Rio Grande do Sul, Porto Alegre, RS, Brazil; 4 Institute of Nutrition, Friedrich Schiller University Jena, Jena, Germany

## Abstract

**Background:**

We sought to investigate the relationships among the plasma levels of carotenoids, tocopherols, endogenous antioxidants, oxidative damage and lipid profiles and their possible effects on the cardiovascular risk associated with hemodialysis (HD) patients.

**Methods:**

The study groups were divided into HD and healthy subjects. Plasma carotenoid, tocopherol and malondialdehyde (MDA) levels, as well as erythrocyte reduced glutathione (GSH), were measured by HPLC. Blood antioxidant enzymes, kidney function biomarkers and the lipid profiles were analyzed by spectrophotometric methods.

**Results:**

Plasma lycopene levels and blood glutathione peroxidase (GPx) activity were significantly decreased in HD patients compared with healthy subjects. Total cholesterol, low-density lipoprotein cholesterol (LDL-c), creatinine, urea, MDA, GSH, superoxide dismutase (SOD) and catalase (CAT) were significantly increased in HD (p < 0.05). Lycopene levels were correlated with MDA (r = -0.50; p < 0.01), LDL-c (r = -0.38; p = 0.01) levels, the LDL-c/HDL-c index (r = -0.33; p = 0.03) and GPx activity (r = 0.30; p = 0.03). Regression models showed that lycopene levels were correlated with LDL-c (β estimated = -31.59; p = 0.04), while gender was correlated with the TC/HDL-c index and triglycerides. Age did not present a correlation with the parameters evaluated. GPx activity was negatively correlated with MDA levels and with the LDL-c/HDL-c and CT/HDL-c indexes.

**Conclusions:**

Lycopene may represent an additional factor that contributes to reduced lipid peroxidation and atherogenesis in hemodialysis patients.

## Background

In chronic renal failure (CRF) patients undergoing hemodialysis (HD) treatment, the formation of reactive oxygen species (ROS) is amplified, and oxidative stress may be one of the most relevant complications [1]. The increase in ROS is due to uremic toxins, bio-incompatible dialysis water, non-sterile dialysate, poor quality of dialysis water, back-leak of contaminants across the dialysis membrane and time of hemodialysis treatment [2,3].

In hemodialysis patients (HD), the major cause of death is cardiovascular disease, which is responsible for 50% of the mortality in this population [4,5]. The physiopathology of cardiovascular events in HD is multifactorial, but accelerated atherosclerosis seems to play a central role in cardiovascular dysfunction. In addition, current evidence suggests that the high prevalence of cardiovascular events in these patients is directly linked to oxidative stress and to the abnormality of the plasma lipid profile [6-10].

The abnormalities in the antioxidant defense system and increased oxidative stress may lead to higher susceptibility to lipid peroxidation of low density lipoprotein (LDL) [2,3,11]. Antioxidant vitamins and dietary constituents (for example, vitamin C, tocopherols, α-carotene and other carotenoids) may play an important role in protection against oxidative damage and, consequently, against atherosclerosis [12-14].

Although there are published studies that have evaluated biochemical alterations, endogenous antioxidants and vitamins in HD patients [15], there is lack of information about the relationships among oxidative stress biomarkers, vitamins and classical biochemical alterations. Therefore, the aim of this cross-sectional study was to evaluate whether exogenous and/or endogenous antioxidants may affect lipid peroxidation, cholesterol or cholesterol lipoproteins, which are important biomarkers involved in atherosclerosis in HD patients. Exogenous antioxidants, such as carotenoids (lycopene, lutein, zeaxanthin, α- and β-carotene, β-cryptoxanthin), vitamin E (α- and γ-tocopherol), and endogenous antioxidants (GSH, SOD, GPx and CAT), along with the serum lipid profile, lipid peroxidation biomarkers, and markers of serum kidney function, were evaluated.

## Methods

### Subjects

The study was approved by the Human Ethics Committee of the Science Health Center of the Federal University of Santa Maria (protocol number 091/2003). All patients gave their informed consent prior to inclusion in the study. The study included twenty-nine patients diagnosed with CRF (19 men and 10 women) and submitted to regular hemodialysis treatment at Charity and Health House Hospitals. Additionally, 20 healthy subjects, including 10 men and 10 woman (control group), were enrolled. In order to participate in this research, patients were required to have been receiving regular hemodialysis treatment. Only non-smokers who were free of any problems related to alcoholism, viral hepatitis and HIV were included in the study. Patients using any antioxidant vitamin within the last three months were also excluded. The control group did not have clinical histories of renal diseases or other pathologies. None of the healthy subjects received vitamins. The subjects were all non-smokers and did not consume alcohol regularly.

For the sampling, 10 ml of venous blood was collected from the HD patients before the hemodialysis process (two days after the last process) under conditions of fasting. Blood was also collected from the subjects in the control group. The analysis samples were divided into heparinized tubes, EDTA-containing tubes, and tubes without anticoagulant. Plasma-EDTA and serum were obtained by centrifugation at 1500 *g *for 10 minutes at 4°C. The plasma samples were used to evaluate carotenoids and tocopherols. To assess the antioxidant enzymes, blood samples were stored at -20°C until analysis. Plasma malondialdehyde (MDA) and erythrocyte reduced glutathione (GSH) were processed immediately followed by the determination of hemoglobin (Hb) levels and hematocrit (Hct) in the blood, as well as albumin, iron, ferritin, cholesterol, triglycerides, urea and creatinine levels in the serum.

The quantification of plasma carotenoids (lutein, zeaxanthin, lycopene, β-cryptoxanthin, α- and β-carotene) and tocopherols (α and δ-tocopherol) was carried out after liquid-liquid extraction with a solution of n-butanol:ethanol (50:50) in BHT (2,6-di-ter-butyl-4-methylphenol) mixed by vortexing and followed by centrifugation. The supernatant was analyzed by high performance liquid chromatography (HPLC) with a UV/VIS and fluorescent detector according to Epler et al., 1993 [16] with modifications. The β- apo - 8 carotene was used as an internal standard.

Hemoglobin levels and hematocrit were determined in a Cobas Micros system (Hematology Analyzer, Roche Diagnostics^®^). The biochemistry analyses consisted of albumin, iron, ferritin, serum total cholesterol (TC), lower-density lipoprotein cholesterol (LDL-c), high-density lipoprotein cholesterol (HDL-c), triglycerides, urea and creatinine, which were determined using the Cobas Integra 400 (Roche Diagnostics^®^) automatic device and commercial kits. The indexes of coronary risk were obtained through the following calculations: TC/HDL-c and LDL-c/HDL-c.

Lipid peroxidation was estimated by the measurement of plasmatic malondialdehyde, which was determined by a high performance liquid chromato-graphic system with visible detection (HPLC-VIS) by Knauer^® ^using a method developed in our laboratory [17].

The levels of reduced glutathione in erythrocytes were measured by high performance liquid chromatography with the Knauer^® ^system using a method developed in our laboratory [18].

Enzyme assays were performed in total blood with heparin. Superoxide dismutase (SOD) activity was determined at 480 nm based on its ability to inhibit the autoxidation of adrenaline into adrenochrome at an alkaline pH [19]. Catalase (CAT) activity was determined at 280 nm using H_2_O_2 _as substrate [20]. Glutathione peroxidase (GPx) activity was determined using glutathione reductase and NADPH. The method is based on the oxidation of NADPH, which is measured by a decrease in absorbance at 340 nm [21]. These activities were measured spectrophotometrically using a UV-VIS model Hitachi U-1800^®^.

### Statistical analysis

Statistical computations were performed with the Statistica^® ^6.0 software system (Statsoft Inc., 2001). The results are expressed as the mean ± SEM (standard error of the mean). Comparisons between groups were achieved by Student's *t*-test or the Mann-Whitney test depending on the distribution of the variables. Pearson's or Spearman's rank order linear correlation was used to evaluate the relationships between pairs of variables according to their distribution. Multivariate regression analyses were performed with Jump 5.0.1a (SAS Institute Inc., Cary, NC, USA) to evaluate antioxidants with significant protective effects on the lipid profile (triglycerides, total cholesterol, LDL-c and HDL-c, as well as the LDL-c/HDL-c and TC/HDL-c indexes) and lipid peroxidation. Moreover, the possible influences of age and gender were evaluated, and p < 0.05 (5%) was considered significant.

## Results

The mean length of hemodialysis treatment in the HD patient group was 45.68 ± 7.27 months. The age of the HD patients was significantly higher than that of the control group's patients (51.0 ± 2.17 and 43.15 ± 1.30 years (SEM), respectively). However, after regression analysis (Table 1), it was found that age did not have a significant influence on the parameters evaluated.

**Table 1:** Regression analysis realized to lipid peroxidation, lipid profile and antioxidants.

	LDL-c (R^2^) of model: 0.14		HDL-c (R^2^) of model: 0.67		MDA (R^2^) of model: 0.47		TC/HDL (R^2^) of model: 0.53		LDL/HDL (R^2^) of model: 0.60		TG (R^2^) of model: 0.51	
	*β coeff*	*p*	*β coeff*	*p*	*β coeff*	*p*	*β coeff*	*p*	*β coeff*	*p*	*β coeff*	*p*
**Age**	ns	0.75	ns	0.11	ns	0.10	ns	0.08	ns	0.33	ns	0.35
**Gender**	ns	0.66	ns	0.11	ns	0.08	1.05	0.04	ns	0.10	17.82	0.03
**SOD**	ne	-	ns	0.69	ns	0.45	ne	-	ns	0.92	ns	0.29
**CAT**	ne	-	ns	0.48	ns	0.62	ns	0.70	ns	0.80	ns	0.64
**GPx**	ne	-	3.39	0.003	- 0.33	0.02	- 0.54	0.03	- 0.55	0.001	- 10.16	0.004
**GSH**	ns	-	ns	0.66	ns	0.48	ns	0.48	ns	0.89	ns	0.38
**Lycopene**	- 31.59	0.044	ns	0.23	ns	0.75	ns	0.52	ns	0.20	ns	0.96
**Lutein**	ns	0.67	ns	0.60	ns	0.82	ns	0.51	ns	0.47	ns	0.63

ns: non significant.

ne: non entered in this regression model.

The clinical histories of the HD patients were evaluated, and the causes of renal failure were pyelonephritis and glomerulonephritis (n = 6 or 17.2% of the patients), hypertension (n = 3 or 10.3% of the patients), diabetes (n = 3 or 10.3% of the patients), polycystic kidney disease (n = 1 or 3.5% of the patients), tuberculosis of the urinary tract (n = 2 or 7% of the patients), and, in 37.9% of the patients (n = 11), there was no known cause.

With respect to the therapeutic treatment of the HD patients, it was observed that just six patients received vitamin D, while seventeen patients received erythropoietin, and eleven of those patients also received noripurum (saccharate ferric hydroxide). Moreover, statins were used for reducing lipid levels, and none of the patients received nutritional supplementation or a multivitamin complex. The treatments were chosen and changed by clinicians based on constant monitoring of the laboratorial results and clinical observations.

Serum levels of iron and ferritin in HD patients were 53.04 ± 3.69 mg.dL^-1 ^and 347.84 ± 72.40 ng.mL^-1 ^(SEM), respectively. These values were consistent with the reference values. Additionally, other hematological and biochemical analyses were performed monthly, and the results are presented in Tables 2 and 3 (reference values are also presented). Hematocrit, hemoglobin levels and albumin levels were lower in HD patients than in the control group, and they were below of the reference values. Biomarkers of renal function, including creatinine and urea, were significantly higher in HD patients and were above the reference values. Moreover, correlations between serum creatinine levels and GPx activity (r = -0.67; p < 0.01), as well as serum urea levels and GPx activity (r = -0.77; p < 0.01), were found.

**Table 2:** Hematological and biochemical analysis.

Parameters	Healthy Subjects (n = 20)		Hemodialysis Patients (n = 29)		Reference Values^57^	
	Men (n = 10)	Women (n = 10)	Men (n = 19)	Women (n = 10)	Men	Women
**Hemoglobin (g.dL^-1^)**	14.90 ± 0.50		9.92 ± 0.29^a, c^			
	16.5 ± 0.4	12.8 ± 0.1	10.0 ± 0.4^c^	9.7 ± 0.5^c^	12 - 18	11 - 16
**Hematocrit (%)**	43.20 ± 1.20		30.54 ± 4.73 ^a, c^			
	46.9 ± 0.8	38.0 ± 0.8	30.8 ± 1.2 ^c ^	29.8 ± 1.4^c^	39 - 53	35 - 47
**Albumin (g.dL^-1^)**	3.98 ± 0.06		3.15 ± 0.03 ^a, c^		3.4 - 5.0	
	4.02 ± 0.1	3.9 ± 0.05	3.2 ± 0.03 ^c^	3.1 ± 0.05 ^c^		
**Creatinine (mg. dL^-1^)**	0.68 ± 0.07		10.17 ± 0.62 ^a, b^		0.8 - 1.3	
	0.89 ± 0.06	0.39 ± 0.03	11.5 ± 0.08	8.40 ± 0.83		
**Urea (mg. dL^-1^)**	27.40 ± 1.60		164.55 ± 7.46 ^a, b^		15 - 39	
	28.96 ± 2.55	25.29 ± 1.36	171.10 ± 10.10	160.21 ± 11.73		

The values are expressed as mean ± standard error of the mean (SEM).

^a ^p < 0.05 when compared with healthy subjects; ^b ^Inter gender difference (p < 0.05); ^c ^Below to reference value.

**Table 3:** Lipid profile results of studied groups and reference values.

Parameters	Healthy Subjects (n = 20)		Hemodialysis Patients (n = 29)		Reference Values^57^	
	Men (n = 10)	Women (n = 10)	Men (n = 19)	Women (n = 10)	Men	Women
**TC (mg.dL^-1^)**	147.27 ± 4.48		181.14 ± 5.38^a^		< 200	
	145.9 ± 7.9	148.9 ± 4.1	178.2 ± 6.2	182.7 ± 10.3		
**LDL-c (mg.dL^-1^)**	72.66 ± 4.37		115.89 ± 3.79^a^		< 130	
	69.4 ± 6.5	76.9 ± 5.6	117.5 ± 4.5	111.5 ± 7.0		
**HDL-c (mg.dL^-1^)**	55.90 ± 3.00		28.89 ± 1.92^a^			
	53.0 ± 3.9	61.0 ± 3.9	29.2 ± 2.9	28.7 ± 2.4	> 40	> 45
**TG (mg.dL^-1^)**	90.70 ± 6.40		160.37 ± 9.28^a^		< 150	
	90.7 ± 6.3	85.4 ± 11.0	175.4 ± 11.9	131.9 ± 11.7 ^b^		
**LDL-c/HDL-c**	1.28 ± 0.11		4.48 ± 0.32^a^		< 3.55	
	1.3 ± 0.1	1.2 ± 0.2	4.7 ± 0.5	4.0 ± 0.2		
**TC/HDL-c**	2.68 ± 0.15		7.12 ± 0.55^a^		< 5	
	2.8 ± 0.2	2.5 ± 0.3	7.3 ± 0.9	6.8 ± 0.6 ^b^		

The values are expressed as mean ± standard error of the mean (SEM).

^a ^p < 0.05 when compared with healthy subjects; ^b ^Inter gender difference (p < 0.05).

Abbreviations: TC: cholesterol total; LDL-c: LDL cholesterol; HDL-c: HDL cholesterol; TG: triglycerides.

The analysis of the lipid profile included total cholesterol (TC), LDL-c, HDL-c, triglycerides and the indexes of coronary risk (ICR), and the results are presented in the Table 3. The results are sub-grouped by gender within each group of study. In the control group, no difference was observed among the lipid profiles when comparing women *vs*. men. In the HD group, the triglycerides were significantly higher in men compared to women.

The levels of total cholesterol, LDL-c and triglycerides are within the reference values for both groups studied. However, the Mann-Whitney test revealed that these variables were significantly higher in the HD group compared with the control group. On the other hand, the HDL-c was below the reference values in HD patients, and it was also significantly decreased compared with the control group. The TC/HDL-c index was twice higher in HD compared to healthy subjects, and the LDL-c/HDL-c index was three times higher. Moreover, in the regression analysis, it was found that gender presented an influence on the TC/HDL index (β estimated = 1.05; p = 0.04) and triglyceride levels (β estimated = 17.82; p = 0.03).

Additionally, the clinical history of the HD group was evaluated for two years after the collection of the blood samples. Seven deaths occurred (24.1% of the total) in this period, and cardiovascular diseases (n = 6; 85.7%) was the main cause of death; only one of these deaths (3.5%) was due to a stroke. However, the Mann-Whitney test did not show statistically significant differences among any of the parameters analyzed between the HD patients that died (n = 7) during the present study and those that survived (n = 22). This lack of significant difference may be related to the small number of individuals in the group that died during the study.

The results of our analyses of the oxidative stress biomarkers are presented in Table 4. The plasma MDA and erythrocyte GSH levels were significantly increased in the HD patients compared to healthy subjects (p < 0.01).

**Table 4:** Results of studied groups: antioxidants and lipid peroxidation biomarker.

Biomarkers	Healthy Subjects (n = 20)		Hemodialysis Patients (n = 29)	
	Men (n = 10)	Women (n = 10)	Men (n = 19)	Women (n = 10)
**GSH (μmol.g/Hb)**	6.03 ± 0.26		7.87 ± 0.27^a^	
	5.6 ± 0.2	6.5 ± 0.4	7.8 ± 0.4	7.9 ± 0.4
**CAT (K/mg Hb)**	27.96 ± 4.18		56.99 ± 4.25^a^	
	26.6 ± 6.9	29.3 ± 5.2	55.5 ± 5.5	61.1 ± 7.5
**GPx**	11.57 ± 0.39		6.61 ± 0.21^a^	
**(μmol NADPH.min/g Hb)**	12.2 ± 0.5	11.0 ± 0.5	6.5 ± 0.3	7.0 ± 0.3
**SOD (U SOD/mg Hb)**	0.69 ± 0.49		0.91 ± 0.03^a^	
	0.6 ± 0.04	0.8 ± 0.07	0.9 ± 0.05	0.9 ± 0.05
**MDA (μmol/L)**	4.53 ± 0.16		6.92 ± 0.36^a^	
	4.3 ± 0.2	4.8 ± 0.3	7.4 ± 0.6	6.1 ± 0.2

The values represent the mean ± standard error of the mean (SEM).

^a ^p < 0.05 when compared with healthy subjects.

Abbreviations: GSH: reduced glutathione; CAT: catalase activity; GPX: glutathione peroxidase activity;

SOD: superoxide dismutase activity; MDA: malondialdehyde.

The blood SOD and CAT activities were significantly increased (p < 0.05), while GPx activity was significantly decreased (p < 0.01) in HD patients compared with the control group. Moreover, in the control group, SOD, CAT and GPx activities were similar between women and men. On the other hand, the CAT activity was influenced by gender in the HD group, as it was higher in women than men. The results of the Mann-Whitney test showed that gender had a significant effect on some of the parameters evaluated (Tables 2 to 5). However, only the TC/HDL-c index and triglyceride levels were influenced by gender in regression models (Table 1).

The non-enzymatic and enzymatic endogenous antioxidants listed above were linearly associated the hematocrit and hemoglobin levels, as well as urea and creatinine levels (data no shown). In addition to these correlations, the lipid profile also presented linear associations with endogenous antioxidants (p < 0.05). GSH was significantly associated (p < 0.05) with LDL-c (r = 0.38), HDL-c (r = -0.43), the LDL/HDL index (r = 0.47), the TC/HDL index (r = 0.41) and MDA (r = 0.54). SOD and CAT activities were correlated with HDL-c (r = -0.34 and -0.48), the LDL/HDL index (r = 0.31 and 0.42) and MDA (r = 0.49 and 0.45). In addition, CAT was positively associated with the TC/HDL index (r = 0.52). However, a positive association was observed between GPx activity and HDL (r = 0.64), and a negative association was observed between GPx activity and LDL-c (r = -0.64), the LDL/HDL index (r = -0.72), the TC/HDL index (r = -0.66) and MDA (r = -0.73).

The results of our analyses of carotenoids and total-tocopherols (T-tocopherols) are summarized in Table 5. There was no significant difference for lutein, zeaxanthin, cryptoxanthin or β-carotene levels between the HD group and the control group (p > 0.05). Moreover, both alpha-carotene and lycopene levels were reduced by half in the hemodialysis group compared with the control group (p < 0.05). Additionally, *Spearman's *correlation showed that lycopene levels were negatively correlated with MDA levels (r = -0.50; p < 0.01), LDL-c (r = -0.38; p = 0.01) and the LDL-c/HDL-c index (r = -0.33; p = 0.03). Otherwise, this exogenous antioxidant was positively correlated with GPx activity (r = 0.30; p = 0.03). Furthermore, significant associations (p < 0.05) between lutein and LDL-c (r = -0.30), the LDL/HDL index (r = -0.33) and MDA levels (r = -0.45) were observed.

**Table 5:** Results of vitamin levels analyzed in plasma of subjects studied.

Vitamins	Healthy Subjects (n = 20)		Hemodialysis Patients (n = 29)	
	Men (n = 10)	Women (n = 10)	Men (n = 19)	Women (n = 10)
**Lutein**	0.62 ± 0.06		0.46 ± 0.05	
	0.5 ± 0.05	0.7 ± 0.1	0.5 ± 0.08	0.5 ± 0.05
**Zeaxanthin**	0.08 ± 0.01		0.07 ± 0.01	
	0.07 ± 0.004	0.1 ± 0.01	0.07 ± 0.008	0.07 ± 0.005
**Criptoxanthin**	0.18 ± 0.07		0.21 ± 0.04	
	0.1 ± 0.02	0.2 ± 0.1	0.1 ± 0.03	0.3 ± 0.09
**α-Carotene**	0.18 ± 0.03		0.07 ± 0.01^a^	
	0.08 ± 0.01	0.3 ± 0.05 ^b^	0.07 ± 0.008	0.09 ± 0.01
**β-Carotene**	0.64 ± 0.09		0.50 ± 0.07	
	0.4 ± 0.05	0.9 ± 0.1^b^	0.4 ± 0.03	0.7 ± 0.2 ^b^
**Lycopene**	0.64 ± 0.06		0.35 ± 0.05^a^	
	0.6 ± 0.06	0.7 ± 0.1	0.3 ± 0.06	0.4 ± 0.1
**T-Tocopherols**	29.6 ± 1.3		33.2 ± 1.9	
	29.8 ± 2.0	29.4 ± 1.8	29.4 ± 1.7	41 ± 3.4 ^b^

The values represent the mean ± standard error of the mean (SEM).

The unit for all parameters is μmol.L^-1^.

^a ^p < 0.05 when compared with healthy subjects.

^b ^Inter-gender difference (p < 0.05).

Regression models (Table 1) were performed based on the significant correlations obtained among exogenous and endogenous antioxidants and the lipid profile and the lipid peroxidation product (MDA). The total cholesterol and triglyceride levels were not associated with other biochemical parameters or with exogenous or endogenous antioxidants except GPx activity. Additionally, hematological and renal biomarkers were entered in these models as co-variables, and they did not influence the interpretation of the models. The results showed that lycopene levels were inversely associated with the LDL-c and HDL-c levels and were positively associated with GPx activity. On the other hand, a negative association was found between GPx activity and MDA levels. Moreover, the GPx activity was a significant variable in the regression models for the TC/HDL-c and LDL-c/HDL-c indexes.

## Discussion

Cardiovascular diseases play an important role in the mortality of HD patients, and we observed that all HD patient deaths that occurred during the study were due to vascular diseases. The pathogenesis of atherosclerotic ischemic heart disease resulted, in part, from the overproduction of reactive species of oxygen and nitrogen. Furthermore, HD process is associated with impairment of the antioxidant defense mechanisms, which is one of the main factors that contributes to cardiovascular disease and is a significant cause of morbidity and mortality in these patients [22].

Therefore, it is of particular interest to explore the possible associations among oxidative stress biomarkers, exogenous antioxidants, such as vitamins, and, especially, the carotenoids, the lipid profile and the risk of cardiovascular disease in HD patients.

In the present study, HD patients showed a moderate increase in triglycerides and total and LDL cholesterol (TC and LDL-c), as well as a decrease in high-density lipoprotein (HDL) cholesterol, and these findings were in accordance with those of other authors [23]. The elevated levels of LDL cholesterol may provide increased substrates for reactive oxygen species. These free radicals react with the low-density lipoprotein (LDL), leading to the formation of oxidized LDL particles, which are important in the initiation and progression of atherosclerotic plaques because they can elicit inflammatory processes and lipid accumulation within the arterial wall [24]. In addition, Kinosian et al. [25] has reported that the LDL-c/HDL-c and TC/HDL-c indexes are more reliable for assessing the risks for atherogenic diseases than the lipid profile comprised of isolated parameters. The atherogenic risks, including the LDL/HDL and TC/HDL indexes, were elevated in HD patients, suggesting that metabolic abnormalities are present in HD patients that can contribute to cardiovascular disease.

In accordance with previous studies [26,27], HD patients present a vulnerability to oxidative species that is evidenced by elevated levels of MDA. Lipid peroxidation is thought to be the main factor influencing atherogenesis [28,29]. Moreover, the multivariate analysis demonstrated that the increase of lipid peroxidation was negatively correlated with GPx activity deficiency, demonstrating that ROS generation can contribute to decreased GPx activity.

The results of this study indicate that patients who periodically perform hemodialysis undergo changes to their endogenous antioxidant system. A major finding was the decrease of GPx activity, which was reduced by almost 50 percent in HD patients compared to controls. This decrease in GPx activity may represent an early consequence of active nephron mass reduction, reinforcing the suggestion that the renal tubule is the predominant site of synthesis of GPx [30]. Interestingly, urea and creatinine levels were negatively correlated with GPx activity. Thus, in HD patients, when renal impairment is increased, GPx activity is lower, and, consequently, there is a reduced ability to detoxify hydrogen peroxide, as GPx is considered to be the primary defense for elimination of these reactive species in erythrocytes [31].

In HD patients, the elevation of GSH levels did not seem to be sufficient to protect against the lipid damage because the GPx activity was decreased. It is known that GSH levels are dependent on GPx activity to counter the oxidative imbalance [8]. The GSH elevation in HD patients is known to be a compensation mechanism [26,28,32]. Additionally, the regression models suggest that the elevation of MDA, as well as increases in the TC/HDL and LDL/HDL indexes, could be the most important of the factors analyzed in this study that are associated with GPx deficiency. By contrast, the antioxidant function of HDL-c, in association with antioxidant enzymes [33], is observed to be based on the positive relationship between this cholesterol fraction and GPx activity. However, the lower levels of HDL and GPx activity cannot counter the oxidative [34-37] stress or the increase in atherogenic indexes. Moreover, CAT and SOD activities were increased, as well as the GSH levels, to compensate for the oxidative stress that results from the hemodialysis process [30,38].

With respect to exogenous antioxidants, the antioxidant and anti-inflammatory activity [8] of vitamin E (α- and γ-tocopherol) is known, and the vitamin E deficiency in HD patients conflicts with previous results [39]. In this work, the plasma vitamin E levels were not reduced HD patients in comparison with controls, which is consistent with previous studies where blood collection was performed before hemodialysis treatment [40,41]. Moreover, the levels of vitamin E were not associated with their main function, which is to prevent lipid peroxidation and, consequently, to prevent LDL oxidation [42,43]. However, this lack of association could be due to the fact that we evaluated only MDA levels, and the specific LDL oxidation product was not quantified.

The majority of the previous studies evaluated the total carotenoids [44] or β-carotene [45]; in the present study, we investigated the individual carotenoids. It is known that carotenoids have an important role in the prevention of human diseases, such as cataracts, cancer and other diseases acting as antioxidants [38,46], as well as heart protection in hemodialysis patients [47]. Usually, dialysis patients have abnormal levels of vitamin and carotenoid because of inadequate dietary intake resulting from poor appetite, dietary restrictions and metabolic disorders associated with renal failure [47,48]. In this study, the HD patients only had lower levels of lycopene and α-carotene in comparison to controls. Several studies have indicated that lycopene is an effective antioxidant and a free radical scavenger because of the number of conjugated double bonds [46,49]. Lycopene has also been suggested as a possible carcinogenesis and atherogenesis prevention agent because it protects critical biomolecules, including lipids, low-density lipoproteins (LDL), proteins and DNA [50]. Our results are consistent with previous reports [30,51], which found that levels of lycopene are reduced almost by half in HD patients compared with healthy subjects. In 1994, Loughrey et al. [52] demonstrated that macrophage enrichment with lycopene or with α-carotene results in the suppression of cellular cholesterol synthesis and an increase in macrophage LDL receptor activity. This effect can lead to enhanced clearance of LDL from the plasma. In this line, the multivariate analysis conducted here demonstrated an inverse association of lycopene levels and LDL-c levels, suggesting that lycopene could be considered an important extrinsic factor for the prevention of atherosclerosis, which is consistent with the findings of Rissanen et al., 2002 [53]. In addition to a probable protective effect of lycopene, these results demonstrated a lack of association with gender or age.

In the present study, we found that α-carotene levels were higher in the control group compared with healthy subjects in a Canadian study [51]. This could be due to interregional variations in diet. Levels of β-carotene, lutein, zeaxanthin and β-cryptoxanthin showed no significant differences between the studied groups. Levels of β-cryptoxanthin and β-carotene were consistent with those reported by Sundl et al. 2009 [51]. Moreover, an influence of gender on the levels of these carotenoids was also observed in another study, which reported that the values were higher in women than in men [54]. Interestingly, there are studies that report the existence of the reference values for different populations [55]. Our results are similar to those obtained in a Spanish population [56]; however, in this population, inter-gender variation was not observed. With respect to zeaxanthin and lutein levels, our results are consistent with those of previous reports [51,54].

Thus, our results demonstrated that GPx, an endogenous antioxidant with an important function in renal tissue, influenced lipid damage. Importantly, lycopene, an exogenous antioxidant, may modulate lipid profile disturbance and, potentially, be protective against atherosclerosis in HD patients. Subject age did not influence the evaluated indexes of cardiovascular risk or the markers of oxidative stress and vitamins. However, gender was found to influence triglycerides and the total cholesterol/HDL-c index.

## Conclusions

We showed that hemodialysis patients had reduced of lycopene levels and GPx enzyme activity which were associated with an increase in lipid damage that was evident based on increased lipid peroxidation and decreased HDL-c levels. It was demonstrated that the HD patients have higher index values for atherogenic risk. Thus, the decline of important exogenous (lycopene) and endogenous (GPx activity) antioxidants associated with the lipid profile could directly influence morbidity and mortality in these patients. However, more studies are required to verify whether it is possible to increase lycopene levels through diet, and the likely role of decreased GPx activity in cardiac risk and atherogenesis in this group of patients should be further investigated.

## Competing interests

The authors declare that they have no competing interests.

## Authors' contributions

The authors MR, JV, CP, AM, MC, RB, NB, FA, MD, ML have made substantial contributions to the conception and design of the study, the acquisition of data and the analysis and interpretation of data, and the authors TG and SG participated in drafting the manuscript or critically revising it for important intellectual content. The authors have also given final approval of the version to be published. Each author has participated sufficiently in the work to take public responsibility for appropriate sections of the content.

## Pre-publication history

The pre-publication history for this paper can be accessed here:

http://www.biomedcentral.com/1471-2369/12/59/prepub
